# Towards A Network of Locally Managed Marine Areas (LMMAs) in the Western Indian Ocean

**DOI:** 10.1371/journal.pone.0103000

**Published:** 2014-07-23

**Authors:** Steve Rocliffe, Shawn Peabody, Melita Samoilys, Julie P. Hawkins

**Affiliations:** 1 Environment Department, University of York, York, United Kingdom; 2 Blue Ventures Conservation, London, United Kingdom; 3 CORDIO East Africa, Mombasa, Kenya; University of California- Santa Barbara, United States of America

## Abstract

In the Western Indian Ocean (WIO), local communities are increasingly assuming responsibility for inshore marine resources either on their own or through collaborative management arrangements with governments or non-state actors. In this paper, we trace the evolution and expansion of community management in the WIO and present the first ever inventory and assessment of the region’s locally managed marine areas (LMMAs). We compare the key attributes of these areas to those under government stewardship and assess their relative contributions to progress towards the Convention on Biodiversity (CBD) target of 10% of marine and coastal ecological regions to be effectively conserved by 2020. We also explore the legal frameworks that underpin locally managed marine initiatives in Kenya, Madagascar, Mozambique and Tanzania to assess the potential for future expansion. A principal finding is that whilst LMMAs protect more than 11,000 square kilometres of marine resource in the WIO, they are hampered by underdeveloped local and national legal structures and enforcement mechanisms. In our recommendations to improve local management, we suggest establishing a network of LMMA practitioners in the WIO region to share experiences and best practice.

## Introduction

Despite their value to humans, marine ecosystems worldwide are threatened by a range of anthropogenic pressures, including pollution, habitat loss, climate change and overfishing [1]–[3]. These impacts have drained populations of culturally and economically important fish stocks and reduced structural complexity of various marine communities across a rich range of habitats, species and trophic levels [3]–[5].

In the Western Indian Ocean (WIO) as throughout the world, Marine Protected Areas (MPAs) have been a primary management approach in attempts to alleviate anthropogenic pressures [6]. An MPA is defined by IUCN as: “A clearly defined geographical space, recognised, dedicated and managed, through legal or other effective means, to achieve the long-term conservation of nature with associated ecosystem services and cultural values.” [7]. Solid evidence from MPAs, particularly for No-take Zones (MPAs that allow no extraction), shows that protection can increase average size, diversity, abundance and biomass of species [3], [8], [9] and that some of this biomass can be exported beyond protected boundaries [10]–[12]. MPAs can also play an important role in climate change adaptation, enhancing ecosystem resilience and protecting vital ecosystem services [13], [14].

In 2002, international leaders at the World Summit on Sustainable Development set the first target for the establishment of a global system of MPAs [15]. This target was formally quantified four years later, when the parties to the Convention on Biological Diversity (CBD) committed to effectively conserving 10% of each of the world’s ecological regions by 2012 [16]. In 2010, the parties pushed back the deadline to 2020 and adopted Aichi Biodiversity Target 11, with a revised goal of conserving “at least 17 per cent of terrestrial and inland water, and 10 per cent of coastal and marine areas, through effectively and equitably managed, ecologically representative and well connected systems of protected areas and other effective area-based conservation measures” [17].

Initial progress towards these targets was slow: based on the rate of MPA expansion to 2008, Wood estimated that the 10 percent figure would not be achieved until 2047 [18]. In contrast, the most recent analysis [19], paints a more optimistic picture. MPA coverage has increased dramatically, quadrupling between 2002 and 2012 (*ibid.*). MPAs now cover 8.3 million km^2^, 2.3% of the global ocean area and 7.9% of the continental shelf and equivalent areas (i.e. less than 200 m deep) (*ibid.*). So pronounced is the increase that the 10 percent Aichi CBD target could be reached, even before 2020 (*ibid.*). However, a few very large MPAs are largely responsible for this apparent reversal of fortunes, a trend that looks set to continue as new super-sized protected areas come online in Cook Islands and New Caledonian waters [19], [20]. Further, most of these MPAs are located in uninhabited or low-population-density areas [19] and/or in developing countries where enforcement is weak to non-existent [21].

Although their popularity continues to increase, marine protected areas often fall short of their original goals and sometimes fail entirely, though published negative evaluations are rare. Inadequate long-term funding and widespread management failure have resulted in unenforceable and ineffectual “paper parks” [22]. The most recent global evaluations suggest that less than 16% of MPA managers feel they have adequate funding for effective conservation [23] and that just 15% of coral reef MPAs are effectively managed [24]. Regional evaluations have reached similar conclusions. In a recent review of marine conservation successes in the WIO, for example, Samoilys & Obura [25] only mention one example of successful government-established MPAs: those of Kenya.

### Locally managed marine areas

As a result of disappointment with top-down, centralised government interventions, and facilitated by increasing recognition of the relative strength of local institutions [26]–[28] local communities throughout the Indo-Pacific are increasingly assuming responsibility for inshore marine resources through collaborative partnerships with governments and/or non-state actors [29]–[32]. In the Pacific, areas where marine resources are at least in part under community control are usually termed “locally managed marine areas” (LMMAs) [33]. In the WIO, the terms used vary more widely, encompassing not only LMMA, but also Collaborative Fisheries Management Area and Community Conservation Area, as well as local names such as *tengefu*, *hifadhi za kijamii* and *vilindo vya wenyeji* in Kenya [25], [34]. Although the level of community involvement and the overall management model is context-specific to an extent, a key aspect is local control. Technical support may be provided by government agencies, private sector stakeholders or non-governmental organisations, but it is the resource users themselves who make most of the management decisions, including the location of any protected areas [35]–[37].

Despite its relatively recent popularity, the approach of managing and conserving marine resources at the local level is actually centuries old [28], [38], [39]. In many tropical nations, especially in Pacific Island countries, informal systems of community marine management were in place prior to colonialism [38], [39]. Further, the tradition of customary marine tenure (CMT) – the right to control access to local fishing grounds – in Pacific Island countries provided an ideal socio-cultural platform on which modern day LMMAs could evolve [40]. However, although there are sacred coastal sites that are protected for spiritual reasons in places like Kenya and Tanzania [41], [42] as well as several taboos around fishing [43], there is no tradition of CMT and this may partly explain why the establishment of LMMAs is a more recent phenomenon in the region.

In the Pacific, more than 500 communities in 15 countries manage 12,000 km^2^ of coastal resources, 1,000 km^2^ of which constitutes full no-take protection [33]. In the Western Indian Ocean, little is known about the status or extent of LMMAs. In this paper, we present the first inventory of LMMAs in the WIO and assess them in terms of geography, numbers, size and governance structures. We compare the key attributes of these areas to those under government stewardship and evaluate potential contributions to international biodiversity commitments. To determine prospects for future LMMA expansion, we also explore the legal frameworks that underpin locally managed marine initiatives in Madagascar, Kenya, Mozambique and Tanzania. Finally, we make recommendations for improving local marine management, including the establishment of a regional network of practitioners to facilitate the sharing of experiences and best practice.

## Methods

### Locally managed marine areas: a definition

Following Govan et al [44], we define a locally managed marine area as *“An area of nearshore waters and coastal resources that is largely or wholly managed at a local level by the coastal communities, land-owning groups, partner organizations, and/or collaborative government representatives who reside or are based in the immediate area.”* Under this definition, LMMAs are managed for sustainable use rather than for conservation per se [24]. Many LMMAs employ a combination of management techniques, including periodic closures, gear restrictions, species specific reserves and permanent fully protected (closed) no-take zones [45], [46].

This wide variety of approaches and focus on sustainable use has lead some to question whether LMMAs should qualify as protected areas and thereby count towards international biodiversity targets [47]. For the IUCN, for example, “only those areas where the main objective is conserving nature can be considered protected areas; this can include many areas with other goals as well, at the same level, but in the case of conflict, nature conservation will be the priority” [7].

Others [44], [48], notably the CBD, are perhaps mindful of the informal nature of many LMMAs and less concerned about the need for an overarching conservation objective. By assuming that LMMAs can help WIO nations to meet their CBD obligations, this second approach is the one we adopt here.

Based on categories suggested by Sen and Raakjaer Nielsen [49] we developed a typology that classifies sites in the WIO along a four-point spectrum according to the extent to which resource management is shared between government and user groups.

#### Level 1: *Central*:

Governments or non-state actors designate and manage the area. No mechanisms exist for dialogue with users and decisions are taken by resource managers.

#### Level 2: *Consultative*:

Governments or partner organisations designate and manage the area. Whilst mechanisms exist for dialogue with users, in practice, most decisions are taken by resource managers.

#### Level 3: *Cooperative*:

Local communities and governments or non-state actors cooperate together as equal partners in decision making.

#### Level 4: *Local*:

In this type of arrangement, government has delegated management authority to local communities. The remit of government or partner organisations is largely restricted to providing advice and endorsing management decisions made by local communities.

For this paper, we classified level 3 and 4 sites as LMMAs and level 1 and 2 sites as MPAs. For the sake of clarity, we refer to the four levels collectively as Marine Managed Areas (herein MMAs) [44].

### Study Site

**Figure 1:**
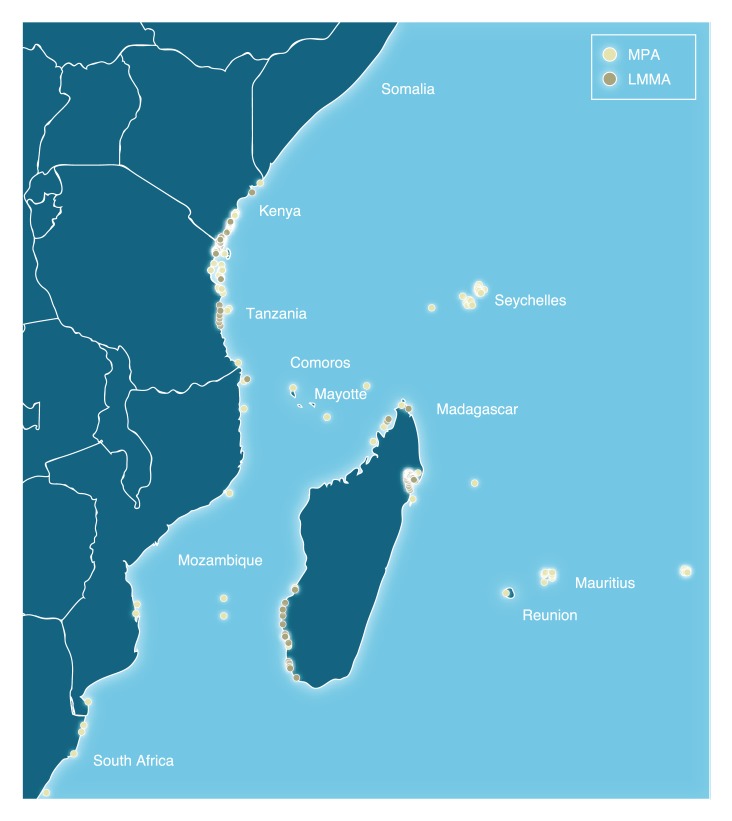
LMMAS and MPAs in the Western Indian Ocean.

The Western Indian Ocean region refers to the African coastal states of Somalia, Kenya, Tanzania, Mozambique and South Africa, together with the Indian Ocean island states of Comoros, Madagascar, Mauritius and Seychelles, as well as the two French overseas departments Mayotte and Réunion (Figure 1). Basic geographic and socio-economic information for the region is summarised in Table 1.

**Table 1:** Geographic scope, population and socio-economic characteristics of Western Indian Ocean (WIO) nations.

Country	Area(km^2^)^1^	Coastline(km)	Pop. (m)2010	% Coastalpop. 2000^2^	Pop.density km^2^	GDP 2010(US$bn)	GNI per cap.2010 (US$)^3^	HDI*2011
Comoros	1,860	340	0.73	100	395	0.54	750	0.433
Kenya	569,140	536	40.51	7.7	71.2	31.41	790	0.509
Madagascar	581,540	4,828	20.71	50.5	35.6	8.72	430	0.48
Mayotte^4^	374	-	0.19	100	-	-	-	-
Mauritius	2,030	372	1.28	100	631.1	9.73	7,750	0.728
Mozambique	786,380	2,470	23.39	55.9	29.7	9.59	440	0.322
Réunion^5^	2,513	-	0.83	100	331.7	-	-	-
Seychelles	460	491	0.09	100	188.1	0.94	9,760	0.773
Somalia	627,340	3,025	9.33	62.7	14.9	-	-	-
South Africa^6^	94,361	-	10.27	37.3	108.8	57.46	6,090	0.619
Tanzania	885,800	1,424	44.84	19.8	50.6	23.06	530	0.466

Source: The World Bank [120], except for coastal population and HDI, which are provided respectively by CIESIN [121] and UNDP [122].

Note: South Africa only includes KwaZulu Natal as the Province that borders the WIO.

*HDI = Human development index (http://hdr.undp.org/en/statistics/indices/hpi/).

1 Excludes area under inland water bodies, national claims to continental shelf, and exclusive economic zones.

2 Percentage of population living within 100 km of a coastline.

3 Gross national income, calculated using World Bank Atlas Method.

4 Figures for Mayotte are calculated from INSEE [123]. Population estimate from 2009.

5 Figures for Réunion are calculated from INSEE [124].

6 South Africa only includes KwaZulu Natal, the Province that borders the WIO. Area and population figures from Statistics South Africa [125]; GDP figures calculated from Statistics South Africa [126].

The mainland WIO area stretches for 13,000 km along the coast from Somalia in the north to South Africa in the south. The island states consist of more than 400 islets and islands with a combined coastline of 6,360 km. The region is ecologically and socio-economically diverse. Overall species composition is rich, exceeding 11,000 species of marine flora and fauna, 60–70% of which are endemic to the Indo-Pacific ocean [50],[51]. There are at least 369 species of coral, 10 mangrove and 12 seagrass, 2,200 coastal fishes, 3,000 molluscs, 450 crabs, 400 echinoderms and five of the world’s seven marine turtle species [50]–[52].

The WIO region has a population of around 152 million people, of which approximately 48.3 million (31.8%) live within 100 km of the coast (Table 1). Population density is diverse, ranging from 15 people per square kilometre in Somalia to 395 in Comoros and 631 in Mauritius. Economically, excluding the French territories (Mayotte, Réunion), GNI per capita totals in the island nations are significantly higher than on the mainland, with figures from the Seychelles more than 12 times those of Kenya and more than 18 times those of Tanzania, two of the largest economies on the mainland. The higher level of socio-economic development in Mauritius and the Seychelles is also underscored by human development index scores, with Seychelles ranked 52 in the world and Mauritius 77–91 places higher than Kenya, which is ranked third in the WIO region.

### Data Compilation

To gather data on level 1 and 2 sites, we synthesised a list of MPAs in the eleven territories under consideration from the academic literature, government agencies, non-government and intergovernmental organisations’ reports, and from the World Database on Protected Areas. We captured information on IUCN category, size and age.

To calculate threat levels to marine resources and MPA effectiveness, we undertook a region-specific re-analysis of global spatial data from Burke et al. [24]’s *Reefs at Risk Revisited,* thus confining ourselves to coral reef habitats. We extracted data on reef extent, reef threats, MPA extent and MPA effectiveness for each nation (one province for South Africa) in the WIO. The MPA effectiveness data for the Western Indian Ocean in Burke et al. [24] cover 59 coral reef MPAs, 66% of the MPAs we documented in this analysis. Because effectiveness was determined through a rapid review using scores from regional experts rather than from field practitioners, there may be a sampling bias toward better-known sites, with a potentially higher proportion of ecologically effective sites than would be found overall (*ibid.*). Where one or more of the authors of this study had in-depth and more recent experience of one of the sites, these ratings were updated where necessary (n = 6) to give a more accurate picture. All spatial modelling was performed with the ArcGIS™ 10.1 Geographic Information System software, and the ArcGIS™ Spatial Analyst extension.

To assess contributions towards international biodiversity commitments, we measured the progress of each country, except France, towards achieving the Convention on Biological Diversity’s (CBD) target of 10% of marine and coastal ecological regions to be effectively conserved by 2020 [17]. Progress was assessed by calculating the percentage coverage by MPAs and LMMAs of the continental shelf to 200 m depth after Wells et al. (2007) [53].

The list of level 3 and 4 sites, the LMMAs, was based on information gathered from LMMA workshops at the Seventh WIOMSA (Western Indian Ocean Marine Science Association) Scientific Symposium, held in Mombasa, Kenya in October 2011 and the Madagascar LMMA Forum, held in June 2012 in Madagascar. The initial workshop outputs were supplemented by an extensive electronic search for published literature using several electronic databases (including Web of Science, ScienceDirect, EconLit, WorldFish Library Catalog and CAB Direct). To capture potential LMMA sites documented in grey literature, we used Google.com and Google Scholar and examined the first 100 hits from each of our searches. Criteria for inclusion of an LMMA in the final list were that its area under management had been formalised through some form of legislation, usually a by-law. We combined the outputs of the workshop and literature searches to create our inventory of a total of 136 MMAs in the Western Indian Ocean and liaised with key individuals and officials to determine or verify locations, sizes and governance structures of these areas.

## Results And Discussion

### Mpas In The Western Indian Ocean

Formally recognised MPAs have been around in the WIO since 1965, when the Ilhas da Inhaca e dos Portugueses Faunal Reserve (now part of the Ponta do Ouro Partial Marine Reserve) was gazetted in Mozambique [54]. Both Madagascar and Kenya followed suit over the next three years, establishing Nosy Tanikely, the Malindi and Watumu Marine National Parks and the Malindi-Watumu Marine National Reserve [54]. Today, all WIO countries have gazetted MPAs, except for Somalia, where the lack of a central administration has made it very difficult to practice conservation [55],[56].

The 74 MPAs identified have a total coverage of 133,273 km^2^. Early protected areas tended to be smaller than their contemporary counterparts and were often designed to protect a specific habitat such as a turtle-nesting beach [53]. Indeed, the smallest MPA in the WIO, the 0.01 km^2^ Cousin Island Special Reserve in the Seychelles dates from 1975. Table 2 shows how the average size of protected areas in the region increased by a factor of six from 49.1 km^2^ between 1965–1974 to 292.4 km^2^ between 1995–2004 as the emphasis shifted to larger, zoned multiple-use sites such as the Quirimbas and Bazaruto Archipelago National Parks in Mozambique. The two largest MPAs in the region are also among the newest: the Marine Park of the Glorieuses, designated in February 2012, and the neighbouring Marine Park of Mayotte, designated two years previously (Personal communication, Pascale Chabanet). The combined area of these new parks alone is more than 100,000 km^2^, constituting more than 84% of the total MPA coverage in the WIO region. This trend towards larger marine protected areas is continuing, with the newly designated Primeiras and Segundas MPA in Mozambique covering over 10,000 km^2^ [57]. Although declarations of large MPAs represent a step forward in marine biodiversity conservation, knowledge of the effectiveness of these areas is needed to properly assess achievements in addressing the Aichi CBD targets.

**Table 2:** Mean area of MPAs in the Western Indian Ocean and numbers existing by decadal creation date.

Year created	Number ofMPAs created	Averagesize (km^2^)
1965–1974	7	49.1
1975–1984	16	36.5
1985–1994	10	25.3
1995–2004	26	292.4
2005–2014	15	8920.8

### Effectiveness Of Protection Of Mpas

Even with the large increase in coverage afforded by the Mayotte and Glorieuses reserves, the 74 MPAs (outlined in Table S1) protect just 7.3% of the continental shelf in the region (Table 3).

**Table 3:** Extent of MPA coverage and effectiveness for coral reef MPAs in the Western Indian Ocean.

Region/Country	Continentalshelf (km^2^)^1^	MPAs (km^2^) inshelf area	MPAcoverage (%)^2^	EffectiveMPAs (%)^3^	Reef Area(km^2^)	Reef area as %of global	Overfishedreefs (%)^4^
Global	24,285,959.0			15.0	249,713.0	100.0	55.0
Indian Ocean				29.0	31,543.0	13.0	60.0
Comoros	1,415.7	404.0	28.5	0.0	399.0	0.2	100.0
Kenya	8,460.1	835.3	9.9	55.6	620.0	0.2	93.8
Madagascar	96,652.8	2603.3	2.7	100.0	3,934.0	1.6	86.9
Mauritius	27,372.8	139.2	0.5	28.6	976.3	0.4	62.6
Mayotte	1,100.0	1,100.0	100.0	0.0	643.8	0.3	78.1
Mozambique	73,299.7	14,551.3	19.9	0.0	2,435.5	1.0	78.5
Réunion	965.1	35.0	3.6	0.0	27.5	0.0	100.0
Seychelles	31,478.7	201.8	0.6	30.0	1,904.3	0.8	9.6
Somalia	40,391.7	0.0	0.0	NA	546.5	0.2	100.0
South Africa^6^	16,093.8	960.9	6.0	50.0	5.0	0.0	100.0
Tanzania^7^	8,951.6	1,160.8	13.0	0.0	2,089.0	0.8	94.7
Tanzania –Zanzibar	8,951.6	1,000.5	11.2	25.0	922.0	0.4	99.7
**WIO** *	**315,133.6**	**22,185.8**	**7.0**	**29.6**	**14,502.8**	**5.8**	**76.4**

1 Source: World Resources Institute [127].

2 Percentage of continental shelf within marine protected areas.

3 Percentage of coral reef MPAs judged to be effective in a rapid appraisal by regional experts. Developed from Burke et al. [24]. Both the Burke analysis and this one use the same definition of an MPA.

4 Percentage of reefs at medium or high risk from overfishing and destructive fishing. Developed from Burke et al. [24].

5 At 68,332 km^2^, Mayotte Marine Park is much larger than its continental shelf area. Until 31 March 2011, Mayotte was not an overseas department of France and separate figures for continental shelf were not attainable. The figure used as a conservative proxy is the size of its lagoon [123].

6 South Africa only includes KwaZulu Natal, the Province that borders the WIO. As continental shelf values for the province weren’t available, a conservative proxy of 10% of South Africa’s total shelf area was used.

7 Figures for continental shelf of the mainland and Zanzibar were unavailable, so an arbitrary split of 50% of Tanzania’s total shelf was used.

*WIO values are totals.

Seventy six percent of reefs in the WIO are at risk from local threats, with half (49.7%) rated at high or very high risk. The problem is most acute in the mainland coasts of Somalia and Tanzania and the islands of Réunion and Comoros, where more than 90% of reefs are threatened. The largest single threat is overfishing, which affects 72% of coral reefs, particularly on the densely populated coastlines of southern Kenya and Tanzania (Table 3). Watershed-based pollution is a problem in places like Madagascar, where widespread deforestation has caused extensive erosion and siltation in coastal areas [24]. Dynamite fishing is also an issue, primarily on the mainland Tanzania coast where it has occurred for decades [25],[58], but it also occurs in Mohéli Marine Park in Comoros (MS pers. obs. 2010).

Only 29.6% of reef-related MPAs assessed in the region were found to be effective. Whilst the effectiveness of MPAs within the WIO appears to range from 0–100% effective, it is important to recognise that artefacts occur at both extremes. For example, Madagascar’s MPAs receive a 100% rating because only one of the seven MPAs was actually appraised, while Mayotte’s score of 0% predates the establishment of the new marine park in 2010 (Personal communication, Pascale Chabanet).

### Lmmas In The Western Indian Ocean

Four of the eleven nations under consideration have active LMMA projects (Table S2). Unlike the region’s legislated MPAs, the WIO’s LMMAs are newer endeavours. Of the 62 sites identified, 60 (96.8%) were established after the year 2000, in line with the passing of legislation to decentralise marine resources management in Kenya, Tanzania, Mozambique and Madagascar.

The 62 LMMAs for which we obtained reliable estimates of size amount to 11,329 km^2^ in total. These varied across four orders of magnitude: the largest, at 1,966.7 km^2^, is Madagascar’s Ankivonjy; the smallest, at 0.118 km^2^, Mkwakwani/Tradewinds in Kenya. Mean LMMA size was 183 km^2^, with a quarter of sites smaller than 2.12 km^2^ and with a median size of 20.75 km^2^. However, these figures obscure important differences between countries, highlighted in Table 4. For example, Kenya’s 14 nascent LMMAs protect a total of 109.6 km^2^ of marine resource, 37 times less than the 4,096.5 km^2^ under management in Tanzania and 61 times less than Madagascar’s LMMA coverage of 6,635.3 km^2^.

**Table 4:** Extent of MPA (level 1 and 2) and LMMA (level 3 and 4) coverage and current progress towards achieving international biodiversity conservation targets.

Country*	No. ofMPAs	MPAcoverage (%)^1^	No ofLMMAs	LMMA av.Size (km^2^)	LMMAcoverage (%)^2^	LMMA + MPAcoverage (%)^3^
Comoros	1	28.5	0	NA	0.0	28.5
Kenya	9	9.9	14	7.83	1.3	11.2
Madagascar	8	2.7	34	195.16	6.9	9.6
Mauritius	13	0.5	0	NA	0.0	0.5
Mayotte	1	100.0	0		0.0	100.0
Mozambique	6	19.9	1	18	0.0	19.9
Réunion	1	3.6	0	NA	0.0	3.6
Seychelles	14	0.6	0	NA	0.0	0.6
Somalia	0	0.0	0	NA	0.0	0.0
South Africa^4^	4	6.0	0	NA	0.0	6.0
Tanzania	10	13.0	12	341.38	45.8	58.7
Tanzania -- Zanzibar	3	11.2	1	470	5.3	16.4
**WIO** **	**69**	**7.0**	**66**	**182.73**	**3.8**	**10.9**

1 Percentage of continental shelf within marine protected areas (level 1 and 2).

2 Percentage of continental shelf within locally managed marine areas (level 3 and 4).

3 Percentage of continental shelf within marine protected areas and locally managed marine areas.

4 South Africa only includes KwaZulu Natal, the Province that borders the WIO.

*Excludes Îles Éparses.

**All are regional totals, except LMMA av. Size (km^2^) which is a mean.

### Combined Coverage And Progress Towards International Targets

In the Western Indian Ocean, MMAs cover a combined 34,321.4 km^2^ of the continental shelf (10.9% –Table 4). Assuming that percentage of the shelf is an acceptable proxy for the Aichi Biodiversity Target 11 target of 10% of marine and coastal areas protected by 2020, then Comoros, Kenya, and Tanzania have already achieved the target (with respective figures of 28.5%, 11.2% and 37.6%). Mozambique (19.9%) has also achieved the target, primarily due to recent designation of Primeiras and Segundas MPA, whilst Madagascar is on course to do so, should the Barren Isles LMMA be established as scheduled in 2014. In total, LMMAs in the WIO cover 11,329.4 km^2^, 3.6% of the region’s continental shelf. The differences between LMMA and MPA coverage are particularly pronounced in mainland Tanzania and Madagascar, where LMMAs cover 3.5 and 2.6 times more area than MPAs, respectively.

### Locally Managed Marine Areas In The Wio: Legal Context

Locally Managed Marine Areas exist primarily in Kenya, Madagascar, Mozambique and Tanzania. To determine potential for future expansion, here we assess the co-management systems in these four countries by exploring legal context, quantifying successes and identifying barriers to replication (Table 5).

**Table 5:** Key features of LMMA initiatives in the Western Indian Oceans.

Country	Formal LMMAs	LMMA success^1^	LMMA potential^2^	Key local-levelinstitutions	Key enablinglegislation	Local namefor LMMAs
Comoros	No	-	Low-Medium	Village fishingassociations	-	-
Kenya	Yes	Medium	High	BeachManagementUnits (BMUs)	BeachManagementUnit Regulations2007	Community Conservation Areas, *tengefu*, Local Marine Management Areas (also LMMAs)
Madagascar	Yes	High	High	Village and multi-village levelfishingassociations. Village councils(Fokontany),Communes	Gestion LocaleSécurisée(GELOSE),*dina,* Décretd’ApplicationNo 848-05	LMMA, Community Managed Protected Area
Mauritius	No	-	Low	-	-	-
Mayotte	No	-	Low	-	-	-
Mozambique	Yes	Low	Medium	Fishing CommunityCouncils(CCPs – ConselhoComunitário dePescas) andCo-managementCommittees(CCG – Comitéde Co-Gestão)	2003 Regulationon MarineFisheries	-
Réunion	No	-	Low	-	-	-
Seychelles	No	-	Medium-low	Praslin FishersAssociation	-	-
Somalia	No	-	Low	-	-	-
South Africa	No	-	Medium-high	LocalSubsistenceCo-ManagementCommittees	Policy for the SmallScale FisheriesSector in SouthAfrica	Small Scale Fishing Community Area *
Tanzania	Yes	High	High	BeachManagementUnits (BMUs)	2003 Fisheries Actand its principalRegulationsof 2009	Collaborative Management areas, Collaborative Fisheries Management Areas (CFMAs)
Tanzania -- Zanzibar	Yes	Medium	Medium-low	Village FisheriesCommittee(VFC), VillageConservationCommittee(VCC)	EnvironmentalManagement forSustainableDevelopment Act1996, The MarineConservation UnitRegulations**	-

1 Level of success in establishing LMMAs to date.

2 Potential to establish more LMMAs in future.

*Forthcoming. Communities will be able to apply several control measures within this area, including quotas and gear restrictions, as well as closed seasons and areas.

**In draft, awaiting finalization.

#### Kenya

Before the recent introduction of Beach Management Units (BMUs) under Legal Notice 402 of the Fisheries Act, marine resource management took a centralised, top-down approach in Kenya [59]. BMUs are a co-management tool for small-scale fisheries initially developed to improve inland fisheries on Lake Victoria [60]. A BMU is an association of fishers, fish traders/mongers, boat owners, fish processors and other fishery stakeholders centred on a coastal landing site and formally led by an executive committee of stakeholders [59],[61]. With the support and permission of officials from the Department of Fisheries, these BMUs are able to devise and enforce by-laws to govern their fishery, allowing them to delineate its boundaries and for example, exclude non-registered fishers or boats from the area [59],[62]. There are presently around 60 BMUs along the Kenyan coastline [61]. Many of these exist only in name, and are yet to formalise their areas of jurisdiction or develop their by-laws [61],[62].

The 2007 BMU Regulations provide a legislative framework to establish Locally Managed Marine Areas in Kenya. Kenya’s first LMMA dates from 2006, when the community of Kuruwitu on the central Kenyan coast established the Kuruwitu Community Managed Conservation Area [63],[64]. Through a local umbrella organisation, the Kuruwitu Community Welfare Association, residents designated a 0.29 km^2^ no-take zone [62],[63]. Since establishment, live hard coral cover within the LMMA has increased by an estimated 30%, whilst fish numbers have grown by 200% [62].

Predating the 2007 BMU Regulations, Kuruwitu lacked legislative support and depended on acceptance from nearby communities and support from the East African Wildlife Society [63],[65]. Nonetheless, the reserve’s success has attracted interest from other fishing communities along the Kenyan coast, and it is likely that Kuruwitu, along with community exchange visits to the Collaborative Management Areas in Tanga, northern Tanzania, helped to catalyse the development of the 2007 BMU regulations and the designation of further LMMAs [25],[62],[63]. At present, there are 14 operational LMMAs in Kenya, covering 110 km^2^ (Table S2).

This rapid increase in LMMA numbers suggests that local communities perceive them to be beneficial, and has occurred in spite of a lengthy development process involving consultations, community surveys, mapping and management plan creation [45]. However, several issues remain, including insufficient capacity for effective monitoring, control and surveillance in local communities, lack of funding and alternative livelihoods, conflicts of interest between stakeholders, legislative overlap and conflicting mandates, and a poor understanding among local communities of the legal procedures involved in designating an LMMA [34],[63],[66]. A further challenge relates to lack of land ownership. In Kuruwitu, for example, the Association hopes to establish a community-run eco-lodge to accommodate visiting tourists, but has so far found it impossible to obtain rights to coastal land on which to construct it [62],[63].

Recent reforms to land policy offer some hope [66]. If these can be combined with guidelines to standardise LMMA establishment, as well as with education and awareness programmes at the local level, then Kenya will be well-placed to lead the LMMA revolution along the coasts of mainland East Africa [63].

#### Tanzania – Mainland

In mainland Tanzania, co-management of marine resources dates back to the mid 1990s, when a collaborative approach was initiated in the coastal waters of the Tanga region [67]. The Tanga Coastal Zone Conservation and Development Programme operated with donor funding from 1994 until 2005 and has continued since as the Tanga Coastal Zone Resources Center, a District and Regional government initiative [68]. Under this Programme six collaborative management areas were established between 1997 and 2001 covering a total of 1,604 km^2^ (Table S2). They have legal recognition in the form of a by-law, as well as formal endorsement from the Director of Fisheries [25],[69].

Each of the Tanga LMMAs has a no-take-zone collaboratively policed by fisheries officers and local communities. Ecological monitoring since 1999 showed that these closures – the first on the East African coast to be established and actively managed by local fishing communities – had higher densities of fish and invertebrates, leading to positive impacts on local livelihoods, at least until 2004 [70],[71]. Since then, dynamite fishing, which was almost completely eradicated in the region between 1998 and 2004, has returned [25].

Mainland Tanzania also has coastal Beach Management Units, established by the 2003 Fisheries Act and its principal Regulations of 2009 [72],[73]. As in Kenya, BMUs empower communities to manage local fisheries resources, giving them the rights to restrict certain gears and control access through licencing [74]. Co-management of fisheries resources has spread rapidly in Tanzania, and there are currently 179 coastal BMUS, of which 68 have management plans and 39 have legal recognition through by-laws [75],[76].

BMUs are increasingly establishing Collaborative Fisheries Management Areas (CFMAs) as a higher-level mechanism to manage their shared resources [76]. CFMAs, a type of LMMA, can protect the fishing grounds of an individual BMU or, more commonly, the shared resources of several [77]. Typically, BMUs with legal recognition first consult with neighbouring Units to determine the boundaries of the CFMA, before establishing a Co-ordination Committee. The Committee, which is composed of representatives from each BMU within the proposed area, synthesises management proposals from individual Units into a draft CFMA management plan, and acts as a networking mechanism for BMUs [77],[78]. Once the draft plan is approved by each participating BMU, the CFMA can be given legal recognition in the form of a District Council by-law. At present, there are six CFMAs in mainland Tanzania, all established with the assistance of the WWF as part of a programme in the Rufiji, Mafia and Kilwa Districts of central Tanzania [34],[75]. The six areas cover a total 2,498 km^2^ across 21 BMUs, 2.5% of which (61.2 km^2^) has no-take protection, initially for a 2-year period [78]. The programme is so far showing some promise: incidences of illegal dynamite fishing and seine netting have decreased, and there is a perception among resource users that fish abundance is starting to recover [79].

#### Tanzania – Zanzibar

BMUs (and CFMAs) do not exist in Zanzibar, a semi-autonomous region of the United Republic of Tanzania with separate environmental law and policy. The most comparable institution is the Village Fisheries Committee (VFC), a local-level organisation comprising 10 elected members [34]. VFCs were formed in all coastal fishing villages when devolution of marine resource management began in 1994 [80]. VFC jurisdiction depends on village boundaries and distance covered by local fishers. Responsibility for enforcement of regulations is shared between the Committee and the Department of Fisheries and VFCs can draw-up by-laws to manage resource use [80],[81].

In Zanzibar, the legal basis for MPA establishment is provided by the Environmental Management for Sustainable Development Act 1996 [82]. The Act recognises the need to involve local communities in MPAs, enabling co-management arrangements analogous to LMMAs to develop at Misali Island and Menai Bay, albeit with a degree of State oversight [53],[81],[83].

In recent years, however, it appears that conservation of marine resources has become more centralised, less transparent and less participatory [84]. For example, Misali Island, a community-initiated attempt to resist tourism development, was subsumed into the larger Pemba Channel Conservation Area in 2005, with a consequential decline in community involvement [50],[83]–[85]. Further, whilst the forthcoming Marine Conservation Unit regulations [86] include provisions to promote community involvement in marine resource management, the overall approach is top-down in nature [84]. These regulations will need a degree of revision if local management is to flourish in Zanzibar.

More broadly, both mainland Tanzania’s BMUs and Zanzibar’s VFCs lack capacity in many crucial areas including conflict resolution, financial management, project planning and marine ecology [34],[75],[79]. There are clear cultural, legal, political and institutional similarities between Kenya and Tanzania, so the efforts that are presently underway in both countries to address capacity constraints and promote sustainable financing would likely benefit from sharing experiences and resources.

#### Madagascar

One of the traditional values recovered following Madagascar’s independence in 1960 was the social code. In rural communities, this social code – known as the *dina* – is a community law, generally communicated through oral tradition, though written down in some cases [87]. In 1996, the Malagasy Government introduced the Gestion Locale Sécurisée (GELOSE), a legal framework designed to integrate the *dina* with governmental laws to enable community-based management of natural resources [87].

Seven years later, at the fifth World Parks Congress in Durban, South Africa, the Malagasy president recognised the need to protect the country’s unique natural assets and committed to the Durban Vision, a national conservation plan to triple the amount of protected area coverage [88]. This was codified into law shortly afterwards as a new decree (Décret d’Application No 848-05) for the existing Code des Aires Protégées [88]. The decree set up a System of Protected Areas of Madagascar, which simplified and redefined the legal process used in protected area creation [89]. Under this more flexible model, community organisations, NGOs and the private sector are permitted to manage protected areas, in addition to the parastatal protected areas agency Madagascar National Parks [90]. Since then, several LMMAs have been established along the coast by NGOs working with local communities (Table S2).

The Velondriake Community Managed Protected Area in southwest Madagascar is the country’s oldest LMMA [91]. Velondriake spans nearly 1,000 km^2^ of coral reefs, mangroves, lagoons, beaches and sea grass beds, making it one of the largest marine managed areas in Madagascar [91],[92]. Home to around 7,500 semi-nomadic Vezo, Velondriake unites 25 coastal villages in the co-management of local marine resources [92],[93]. It is legally recognised as an IUCN category V MPA and was granted definitive protected status by inter-ministerial decree in late 2012 [92]. Velondriake began as an initiative to improve the sustainability of the octopus fishery, but had since expanded to include aquaculture, temporary closures and the designation of eight permanent no-take marine reserves totalling 0.8 km^2^ [92],[93].

The initiative is largely guided and managed by local communities, with technical and financial support provided by the British NGO Blue Ventures. Resource use and access rights within the area are governed by a legally recognised *dina* rather than the GELOSE framework [94]. The *dina* bans destructive fishing practices including beach seining and poison fishing, regulates temporary and permanent closures and grants conflict resolution and enforcement powers to local communities, allowing them to impose fines and utilise the regional court system in cases where conflict resolution is unsuccessful [92],[93].

Velondriake’s perceived success has triggered widespread replication of the LMMA approach. Over the last 7 years, 34 LMMAs have been established along Madagascar’s northern, western and southern coasts. Taken together, these initiatives presently cover 6.9% of the seabed, 6,635.3 km^2^. In 2014, the Barren Islands is expected to add a further 4,290 km^2^ to the total. This scaling up is unparalleled in the Western Indian Ocean, yet it has been achieved at low cost, without financial support from central government [93]. With severe constraints continuing to inhibit the country’s capacity for environmental governance, Madagascar’s LMMAs may offer an encouraging and locally acceptable solution to the challenges of marine resource management [93].

#### Mozambique

In Mozambique, the concept of fisheries co-management is enshrined in the 2003 Regulation on Marine Fisheries, which establishes co-management institutions at the provincial, district and local levels, as well as banning non-artisanal fisheries within three nautical miles of the coast [95]. The Decree introduces two types of institution: Fishing Community Councils (CCPs – Conselho Comunitário de Pescas) and Co-management Committees (CCG – Comité de Co-Gestão) [95]. Both were later formally established through legislation adopted in 2007 [96],[97].

CCGs are multi-stakeholder committees formed principally at the provincial or district levels, but also at the local level [97],[98]. Their principal objectives include deciding closed seasons and permissible types of gear and protecting endangered marine resources as well as advising on conflict resolution among fishers, fishing licences and fee collection [95]. CCPs are community-based associations of elected community members involved in artisanal fisheries [97]. Analogous to Kenya and Tanzania’s BMUs, CCPs give local stakeholders rights to establish boundaries, control access and promote the sustainable use of marine resources [95],[99]. Once members have been elected and the CCP established, they can apply for formal legal recognition, which, if granted, empowers them to assume responsibility for fishing licences and enforcement, functions otherwise administered at the district level [97],[100].

CCP adoption has largely been driven through several donor-led artisanal fisheries programmes, especially in the Sofala Bank area, where the National Institute for the Development of Small-Scale Fisheries and the International Fund for Agricultural Development (IFAD) have worked with local communities to establish 65 Councils along a 950-kilometre stretch of coastline fronting the provinces of Sofala, Zambezia and Nampula [100],[101]. Largely as a result of these initiatives, there are presently at least 156 CCPs along the coastline of Mozambique [102]. Taken together, it is estimated that these Councils provide a degree of representation to almost all coastal fishing communities in the country [103]. However, very few existing councils (approx. 20) are officially recognised by the Ministry of Fisheries [100],[101], whilst many are unaware of their rights and responsibilities, and frequently lack the human resources, technical capacity and financial support necessary for effective management and enforcement [96],[104]. In a study of compliance with centrally declared fisheries controls in the Sofala Bank area, for example, Wilson [104] found that fewer than 10% of inspected nets complied with minimum mesh size regulations, whilst none of the fishers interviewed ceased fishing or reduced effort during the closed season.

Similar technical and financial constraints have also plagued the higher level Co-management Committees. Outside of areas where they have direct support, very few CCGs are functioning, and of those that are, even fewer are endowed with the resources and awareness of community-level rights and obligations they need [96],[98]. So far, there has been little monitoring of CCG operations at the central level and mechanisms for co-ordination across all levels of governance are practically non-existent [98].

As a result of these issues, this study was only able to identify one example of a functioning LMMA in Mozambique: the Vamizi Marine Sanctuary. This no-take reserve is managed by the Vamizi CCP, with technical and financial support from a partnership between an eco-lodge on Vamizi Island and WWF [105]. The partnership is helping to build community capacity for effective marine resource management by training members in reef and fish monitoring, developing alternative livelihood projects and providing environmental education [105]. The Council has delineated the boundaries of the Sanctuary with marker buoys and receives a $3 fee for each dive boat that enters (Personal Communication, Isabel da Silva).

Vamizi’s success suggests if capacity constraints were addressed, more LMMAs could be established. Closed areas have already been trialled in other CCPs, and research suggests that these initiatives, together with restrictions on gear, length and species, may enjoy broad community support in Mozambique, particularly in the Pemba region [106],[107]. To this end, a new phase of the IFAD-financed ProPESCA initiative is aiming to strengthen the capacity of the CCPs and CCGs, whilst the IUCN is considering an intervention to support the creation of community no-take zones [96],[100].

## Conclusions

This analysis shows that although MPAs in the Western Indian Ocean cover 133,273 km^2^, only 7.0% of the region’s continental shelf is protected by them. Less than 30% of reef-related MPAs in the WIO were found to offer effective ecological protection, though this compares favourably with global figures, where 15% were graded effective [24].

We found active LMMA initiatives in four countries that have passed legislation to decentralise marine resource management, with good potential for scaling up these initiatives in Kenya, mainland Tanzania and Madagascar, and lower potential in Zanzibar and Mozambique (Table 5). Due to underdeveloped legal structures supportive of local management in the other seven countries, no other formal LMMA initiatives were found to be in place, though the Seychelles [108],[109], Comoros [110] and especially South Africa [111] have all acknowledged the potential of devolved management.

At 11,329.4 km^2^, LMMAs cover 3.6% of the region’s continental shelf, with particularly pronounced differences between LMMA and MPA coverage in mainland Tanzania and Madagascar, where LMMAs cover 3.5 and 2.6 times more area than MPAs respectively. Assuming that percentage of continental shelf covered by LMMAs and MPAs is an acceptable proxy for the CBD’s 10% 2020 coverage targets, Comoros, Kenya, Mozambique and Tanzania have already achieved the target, whilst Madagascar is on course to do so.

Three caveats apply here. First, LMMA data were based on outputs from a workshop at the Seventh WIOMSA Scientific Symposium in Kenya in October 2011 and the Madagascar LMMA Forum in June 2012 [112] in addition to information gathered from a search of the published and grey literature and first-hand knowledge (MS, SP). Because many LMMAs, especially in Kenya and Mozambique, are small-scale, informal arrangements, information about them can be difficult to source. Accordingly, calculations of LMMA coverage should be considered as conservative estimates.

Secondly, the CBD 2020 targets call for 10% of the world’s ecological regions to be effectively conserved. For the same reasons that the coverage estimates are conservative, the extent to which the region’s LMMAs can be considered effective conservers of resources is largely unknown. Globally, empirical evidence that co-management arrangements achieve ecological [80],[113] and social [32],[36],[114] goals is scarce and inconclusive, though this is likely to be due in part to the recently established nature of many LMMA initiatives. For example, in an examination of 42 marine co-management systems in Kenya, Tanzania, Madagascar, Indonesia and Papua New Guinea, Cinner et al. [115] found that although 88% of resource users surveyed reported high levels of compliance and 54% perceived a benefit to their livelihoods, co-management could also create a degree of social inequality by favouring wealthier users. They additionally found that fish biomass in co-managed areas was generally greater than in areas without local management, though substantially lower than in no-take marine reserves in the same countries [115].

Thirdly, although this analysis suggests that the LMMAs are increasingly numerous and could help WIO nations to meet international biodiversity commitments, we are not necessarily advocating for physically larger LMMAs with greater institutional complexity. Although the perceived success of the Velondriake Community Managed Protected Area in Madagascar provides evidence that large LMMAs with multiple stakeholders can function effectively, [92],[93] other studies have reached differing conclusions. In an examination of ten fisheries cooperatives in Mexico, for example, McCay et al (2014) found that few stakeholders and a small spatial scale were critical success factors in community-based management of the commons [116].

Evidence from a recent global review of Territorial Use Rights for Fisheries (TURFs) by Auriemma et al (2014) is more equivocal [117]. LMMAs and TURFs, the latter defined by the study as “an area in which individuals or communities are given some level of exclusive access to marine resources within a defined boundary”, overlap in many key areas because managed access rights are often implemented by communities within LMMAs [117]. The analysis, which drew on 103 case studies in 29 countries to test, among others, the hypothesis that larger TURFs are less successful due to increasing difficulties of enforcement, found no overall effect [117]. The question of whether bigger LMMAs are better is thus largely unresolved and would benefit from additional research.

From the country-specific analysis of LMMA implementation we present here, it is clear that a lack of organisational capacity, skills and money can all compromise the effectiveness of locally managed marine areas. And where areas are successful, further challenges may arise. For example, as biomass and fish numbers increase, so too may poaching, leading to further strain on resources devoted to enforcement [118],[119].

Over the short-term, these issues may be best addressed through the establishment of an information-exchange forum to enable LMMA practitioners to share experiences and best practice, to offer training and exchange visits, and to promote local management to other communities and governments, especially in countries that have yet to devolve marine resource management to the local level. The forum could be modelled on the Pacific LMMA network and be complemented by research initiatives to better understand under what circumstances LMMAs may achieve their social and ecological objectives. Over the longer-term, the forum could form the basis for scaling-up LMMAs in the region towards a network that is lasting, effective and representative, and one that is complementary to centralised systematic conservation efforts.

## Supporting Information

Table S1 **List of marine protected areas (level 1 and 2) in the Western Indian Ocean.**
(DOCX)

Table S2 **List of locally managed marine areas (level 3 and 4) in the Western Indian Ocean.**
(DOCX)

## References

[1] Halpern BS, Walbridge S, Selkoe KA, Kappel CV, Micheli F, et al (2008) A Global Map of Human Impact on Marine Ecosystems. Science 319: 948–952 10.1126/science.1149345 18276889

[2] Jackson JBC (2008) Ecological extinction and evolution in the brave new ocean. Proc Natl Acad Sci 105: 11458–11465 10.1073/pnas.0802812105 18695220 PMC2556419

[3] Lester S, Halpern B, Grorud-Colvert K, Lubchenco J, Ruttenberg B, et al (2009) Biological effects within no-take marine reserves: a global synthesis. Mar Ecol Prog Ser 384: 33–46 10.3354/meps08029

[4] Fraschetti S, Claudet J, Grorud-Colvert K (2011) Transitioning from single-sector management to ecosystem-based management: what can marine protected areas offer? In: Claudet J, editor. Marine Protected Areas: A Multidisciplinary Approach. Cambridge: Cambridge University Press. 11–34.

[5] Graham NAJ, McClanahan TR, MacNeil MA, Wilson SK, Polunin NVC, et al (2008) Climate Warming, Marine Protected Areas and the Ocean-Scale Integrity of Coral Reef Ecosystems. PLoS ONE 3: e3039 10.1371/journal.pone.0003039 18728776 PMC2516599

[6] IUCN (2004) Managing Marine Protected Areas: A Toolkit for the Western Indian Ocean. Nairobi, Kenya: IUCN Eastern African Regional Programme.

[7] Day J, Dudley N, Hockings M, Holmes G, Laffoley D, et al.. (2012) Guidelines for applying the IUCN Protected Area Management Categories to Marine Protected Areas. Gland, Switzerland: IUCN. 36 p.

[8] Roberts CM, Hawkins J (1997) How small can a marine reserve be and still be effective? Coral Reefs 16: 150–150.

[9] Russ GR, Alcala AC (1996) Marine Reserves: Rates and Patterns of Recovery and Decline of Large Predatory Fish. Ecol Appl 6: 947–961 10.2307/2269497

[10] Gell F, Roberts CM (2003) Benefits beyond boundaries: the fishery effects of marine reserves. Trends Ecol Evol 18: 448–455 10.1016/S0169-5347(03)00189-7

[11] Russ GR, Alcala AC, Maypa AP, Calumpong HP, White AT (2004) Marine reserve benefits local fisheries. Ecol Appl 14: 597–606.

[12] Russ GR, Alcala AC (2010) Enhanced biodiversity beyond marine reserve boundaries: The cup spillith over. Ecol Appl 21: 241–250 10.1890/09-1197.1 21516901

[13] Hastings J, Thomas S, Burgener V, Gjerde K, Laffoley D, et al.. (2012) Safeguarding the Blue Planet: Six Strategies for Accelerating Ocean Protection. Parks 18.

[14] Van Lavieren H, Klaus R (2013) An effective regional Marine Protected Area network for the ROPME Sea Area: Unrealistic vision or realistic possibility? Mar Pollut Bull. doi:10.1016/j.marpolbul.2012.09.004. 10.1016/j.marpolbul.2012.09.004 23294567

[15] World Summit on Sustainable Development (2002) Report of the World Summit on Sustainable Development, Johannesburg, South Africa, 26 August-4 September 2002. UN.

[16] CBD (2006) Report of the Eighth Meeting of the Parties to the Convention on Biological Diversity Curitiba, Brazil: Convention on Biological Diversity.

[17] CBD (2010) Strategic Plan for Biodiversity 2011–2020 and the Aichi Targets. Report of the Tenth Meeting of the Conference of the Parties to the Convention on Biological Diversity. Nagoya, Japan: Convention on Biological Diversity.

[18] Wood LJ, Fish L, Laughren J, Pauly D (2008) Assessing progress towards global marine protection targets: shortfalls in information and action. Oryx 42: 340–351.

[19] Spalding MD, Meliane I, Milam A, Fitzgerald C, Hale LZ (2013) Protecting Marine Spaces: Global Targets and Changing Approaches. Ocean Yearbook 27. Leiden, Netherlands: BRILL.

[20] Pala C (2013) Giant marine reserves pose vast challenges. Science 339: 640–641 10.1126/science.339.6120.640 23393239

[21] Samoilys MA, Martin-Smith KM, Giles BG, Cabrera B, Anticamara JA, et al (2007) Effectiveness of five small Philippines’ coral reef reserves for fish populations depends on site-specific factors, particularly enforcement history. Biol Conserv 136: 584–601 10.1016/j.biocon.2007.01.003

[22] Jennings S (2009) The role of marine protected areas in environmental management. ICES J Mar Sci 66: 16–21 10.1093/icesjms/fsn163

[23] Balmford A, Gravestock P, Hockley N, McClean CJ, Roberts CM (2004) The worldwide costs of marine protected areas. Proc Natl Acad Sci U S A 101: 9694–9697 10.1073/pnas.0403239101 15205483 PMC470737

[24] Burke L, Reytar K, Spalding M, Perry A (2011) Reefs at risk revisited. Washington, D.C: World Resources Institute. 130 p.

[25] Samoilys MA, Obura DO (2011) Marine conservation successes in Eastern Africa. Mombasa, Kenya: CORDIO East Africa.

[26] Christie P (2004) Marine protected areas as biological successes and social failures in Southeast Asia. American Fisheries Society Symposium. Vol. 42: 155–164.

[27] McClanahan TR, Marnane MJ, Cinner JE, Kiene WE (2006) A Comparison of Marine Protected Areas and Alternative Approaches to Coral-Reef Management. Curr Biol 16: 1408–1413 10.1016/j.cub.2006.05.062 16860739

[28] Cinner JE, Wamukota A, Randriamahazo H, Rabearisoa A (2009) Toward institutions for community-based management of inshore marine resources in the Western Indian Ocean. Mar Policy 33: 489–496 10.1016/j.marpol.2008.11.001

[29] Alcala AC, Russ GR (2006) No-take marine reserves and reef fisheries management in the Philippines: a new people power revolution. AMBIO J Hum Environ 35: 245–254. 10.1579/05-a-054r1.1 16989509

[30] Govan H, Tawake A, Tabunakawai K (2006) Community-based marine resource management in the South Pacific. Prot Areas Programme: 63.

[31] Cinner JE, Basurto X, Fidelman P, Kuange J, Lahari R, et al (2012) Institutional designs of customary fisheries management arrangements in Indonesia, Papua New Guinea, and Mexico. Mar Policy 36: 278–285 10.1016/j.marpol.2011.06.005

[32] Wamukota AW, Cinner JE, McClanahan TR (2012) Co-management of coral reef fisheries: A critical evaluation of the literature. Mar Policy 36: 481–488 10.1016/j.marpol.2011.09.001

[33] Govan H (2009) Achieving the potential of locally managed marine areas in the South Pacific. SPC Tradit Mar Resour Manag Knowl Inf Bull 25: 16–25.

[34] Anderson J (2012) Options to Reduce IUU Fishing in Kenya, Tanzania, Uganda and Zanzibar. Ebène, Mauritius: Indian Ocean Commission – SmartFish Program. 99 p.

[35] Tawake A (2007) Scaling-up networks of locally managed marine areas (LMMAs) to island wide ecosystem management while decentralising the effort of Fiji LMMA network and its implementation from national to provincial levels.

[36] Evans L, Cherrett N, Pemsl D (2011) Assessing the impact of fisheries co-management interventions in developing countries: A meta-analysis. J Environ Manage 92: 1938–1949 10.1016/j.jenvman.2011.03.010 21531068

[37] Gutierrez NL, Hilborn R, Defeo O (2011) Leadership, social capital and incentives promote successful fisheries. Nature 470: 386–389 10.1038/nature09689 21209616

[38] Johannes RE (1978) Traditional Marine Conservation Methods in Oceania and their Demise. Annu Rev Ecol Syst 9: 349–364.

[39] Christie P, White AT (1997) Trends in development of coastal area management in tropical countries: From central to community orientation. Coast Manag 25: 155–181 10.1080/08920759709362316

[40] Johannes RE (1981) Words of the lagoon: fishing and marine lore in the Palau District of Micronesia. University of California Press. 280 p.

[41] Burgess ND, Clarke GP, Rodgers WA (1998) Coastal forests of eastern Africa: status, endemism patterns and their potential causes. Biol J Linn Soc 64: 337–367 10.1111/j.1095-8312.1998.tb00337.x

[42] Metcalfe K, Ffrench-Constant R, Gordon I (2010) Sacred sites as hotspots for biodiversity: the Three Sisters Cave complex in coastal Kenya. Oryx 44: 118–123 10.1017/S0030605309990731

[43] Glaesel H (2000) Community-Level Marine Resource Management and the Spirit Realm in Coastal Kenya. Women Nat Resour 21: 35–42.

[44] Govan H, Tawake A (2009) Status and potential of locally-managed marine areas in the South Pacific: meeting nature conservation and sustainable livelihood targets through wide-spread implementation of LMMAs. New Calendonia: Coral Reef Initiatives for the Pacific.

[45] Abunge C (2011) Managing finite marine resources the Kenyan way. Shara: 49.

[46] Mills M, Jupiter SD, Pressey RL, Ban NC, Comley J (2011) Incorporating Effectiveness of Community-Based Management in a National Marine Gap Analysis for Fiji. Conserv Biol 25: 1155–1164 10.1111/j.1523-1739.2011.01749.x 21978136

[47] Govan H, Jupiter S (2013) Can the IUCN 2008 Protected Areas Management Categories Support Pacific Island Approaches to Conservation? Parks 19: 73–80.

[48] Secretariat of the Convention on Biological Diversity (2004) Technical Advice on the Establishment and Management of a National System of Marine and Coastal Protected Areas. SCBD.

[49] Sen S, Raakjaer Nielsen J (1996) Fisheries co-management: a comparative analysis. Mar Policy 20: 405–418.

[50] WWF (2004) Towards the Establishment of an Ecologically Representative Network of Marine Protected Areas in Kenya, Tanzania and Mozambique. Dar es Salaam, Tanzania: WWF Eastern African Marine Ecoregion Programme. 74 p.

[51] UNEP/Nairobi Convention Secretariat (2009) Transboundary Diagnostic Analysis of Land-based Sources and Activities Affecting the Western Indian Ocean Coastal and Marine Environment. Nairobi, Kenya: UNEP. 378 p.

[52] Obura D (2012) The Diversity and Biogeography of Western Indian Ocean Reef-Building Corals. PLoS ONE 7: e45013 10.1371/journal.pone.0045013 23028737 PMC3446983

[53] Wells S, Burgess N, Ngusaru A (2007) Towards the 2012 marine protected area targets in Eastern Africa. Ocean Coast Manag 50: 67–83 10.1016/j.ocecoaman.2006.08.012

[54] UNEP-WCMC (2010) World Database on Protected Areas (WDPA) Annual Release 2010. Cambridge: UNEP-World Conservation Monitoring Centre. Available: http://www.unep-wcmc.org.

[55] IUCN (2000) Progress In Implementing The Jakarta Mandate In The Eastern Africa Region. A Report to the Fifth Conference of the Parties to the Convention on Biological Diversity.

[56] Barrow E, Mahler F, Mosele L, Mvoyi C, Ntahuga L, et al.. (2007) Sound Natural Resource Management: The Foundation for Achieving the MDGs in Somalia. Policy Brief.

[57] MPA News (2012) Mozambique announces coastal protected area. MPA News 14.

[58] Wells S (2009) Dynamite fishing in northern Tanzania-pervasive, problematic and yet preventable. Mar Pollut Bull 58: 20–23. 19056095 10.1016/j.marpolbul.2008.09.019

[59] Government of Kenya (2007) Legal Notice No. 402, The Fisheries (Beach Management Unit) Regulations. The Fisheries Act. Cap 378.

[60] Signa D, Tuda PM, Samoilys M (2008) Social, economic and environmental impacts of beach seining in Kenya.

[61] Lamprey R, Murage DL (2011) Saving our seas: Coast communities and Darwin collaborate for a new future. Swara: 41–48.

[62] Nelson F (2012) Recognition and Support of ICCAs in Kenya. Recognising and Supporting Territories and Areas Conserved By Indigenous Peoples And Local Communities: Global Overview and National Case Studies. Technical Series. Montreal, Canada: Secretariat of the Convention on Biological Diversity, ICCA Consortium, Kalpavriksh, and Natural Justice.

[63] Maina GW, Osuka K, Samoilys M (2011) Opportunities and challenges of community-based marine protected areas in Kenya. Mombasa, Kenya: CORDIO East Afica.

[64] Griffin T (2012) The Kuruwitu Conservation and Welfare Association. People Environ WIOMSA Mag: 13–17.

[65] Murage D (2012) Community Conserved Areas: Experiences from Coastal Communities in Kenya. People Environ WIOMSA Mag: 18–20.

[66] Samoilys M, Osuka K, Maina GW (2011) Opportunities and challenges of current legislation for effective conservation in the Tana Delta-Pate Island coast of Kenya. CORDIO Status Report. Mombasa, Kenya: CORDIO East Africa. p. 16.

[67] Verheij E, Makoloweka S, Kalombo H (2004) Collaborative coastal management improves coral reefs and fisheries in Tanga, Tanzania. Ocean Coast Manag 47: 309–320 10.1016/j.ocecoaman.2004.07.002

[68] Samoilys MA, Kanyange NW (2008) Natural resource dependence, livelihoods and development: Perceptions from Kiunga, Kenya. Nairobi, Kenya: IUCN Eastern Africa Regional Office. 30 p.

[69] Wells S, Makoloweka S, Samoilys M, editors (2007) Putting Adaptive Management Into Practice: Collaborative Coastal Management in Tanga, northern Tanzania. Nairobi, Kenya: IUCN Eastern Africa Regional Office. 197 p.

[70] Samoilys M, Horrill C, Kalombo H, Kabamba J, Wells S (2007) Coral reefs and mangroves: maintaining ecosystem health. Putting Adaptive Management Into Practice: Collaborative Coastal Management in Tanga, northern Tanzania. Nairobi, Kenya: IUCN Eastern Africa Regional Office. 77–102.

[71] Wells S, Samoilys M, Anderson J, Kalombo H, Makoloweka S (2007) Collaborative Fisheries Management in Tanga, Northern Tanzania. Fisheries Management: Progress towards sustainability. Oxford, United Kingdom. 139–165.

[72] United Republic of Tanzania (2003) The Fisheries Act 2003.

[73] United Republic of Tanzania (2009) The Fisheries Regulations 2009.

[74] Mulyila EJ, Matsuoka T, Anraku K (2012) Sustainability of fishers’ communities in tropical island fisheries from the perspectives of resource use and management: a comparative study of Pohnpei (Micronesia), Mafia (Tanzania), and Guimaras (Philippines). Fish Sci 78: 947–964 10.1007/s12562-012-0500-x

[75] Sobo FS (2012) Community Participation in Fisheries Management in Tanzania. IIFET 2012 Tanzania Proceedings. Dar es Salaam, Tanzania. p. 11.

[76] Otsyina RM, Benno BL, Abdallah JM (2010) Strengthening Community Capacity for Fisheries Co-Management (SCCaFCoM) in Rufiji, Mafia and Kilwa Districts: Final Evaluation Report. Dar es Salaam, Tanzania: WWF Tanzania.

[77] Fisheries Development Division, WWF (2009) Guidelines for Establishing Community Based Collaborative Fisheries Management in Marine Waters of Tanzania. Dar es Salaam, Tanzania: Ministry of Livestock and Fisheries Development.

[78] Mwangamilo J (2012) Collaborative Fisheries Management Initiatives. Rufiji-Mafia-Kilwa (rumaki) Seascape Programme.

[79] Tanzania Natural Resource Forum (2012) Community Based Natural Resource Management (CBNRM) Stocktaking Workshop Report.

[80] Cinner JE, Daw TM, McClanahan TR, Muthiga N, Abunge C, et al.. (2012) Transitions toward co-management: The process of marine resource management devolution in three east African countries. Glob Environ Change. doi:10.1016/j.gloenvcha.2012.03.002.

[81] Mwaipopo N (2008) The social dimensions of marine protected areas: a case study of the Mafia Island Marine Park in Tanzania. Dar es Salaam, Tanzania: International Collective in Support of Fishworkers. 54 p.

[82] Government of Zanzibar (2007) The Environmental Management for Sustainable Development Act, 1996. Legal Supplement (Part 1) to the Zanzibar Government Gazette. Vol. CVI No.5743.

[83] Levine A (2007) Staying afloat: State agencies, local communities, and international involvement in marine protected area management in Zanzibar, Tanzania. Conserv Soc 5: 562.

[84] McLean B, Hikmany AN, Mangora M, Shalli M (2012) An Assessment of Legal and Institutional Framework for Effective Management of Marine Managed Areas in Tanzania. Zanzibar Report. Dar es Salaam, Tanzania: Marine Conservation Unit.

[85] Lindhjem H (2003) Sustainable Financing of Marine Protected Areas in Zanzibar. Washington DC: World Bank.

[86] Government of Zanzibar (2012) The Marine Conservation Unit Regulations. Unpublished Draft Regulations.

[87] Rakotoson LR, Tanner K (2006) Community-based governance of coastal zone and marine resources in Madagascar. Ocean Coast Manag 49: 855–872 10.1016/j.ocecoaman.2006.08.003

[88] Durbin J (2007) Madagascar’s new system of protected areas – Implementing the “Durban Vision.” London, UK: Zoological Society of London.

[89] IRIN (2006) Madagascar establishes new park system to protect lemurs, benefit people. IRIN. Available: http://news.mongabay.com/2006/0117-madagascar.html.

[90] Rabearivony J, Thorstrom R, de Roland LA., Rakotondratsima M, Razafimanjato G, et al.. (2010) Protected area surface extension in Madagascar: Do endemism and threatened species remain useful criteria for site selection? Madag Conserv Dev 5.

[91] Harris A (2007) “To live with the Sea” Development of the Velondriake Community-Managed Protected Area Network, Southwest Madagascar. Madag Conserv Dev 2.

[92] Westerman K, Gardner CJ (2013) Adoption of socio-cultural norms to increase community compliance in permanent marine reserves in southwest Madagascar. Conserv Evid: 4–9.

[93] Harris AR (2011) Out of sight but no longer out of mind: a climate of change for marine conservation in Madagascar. Madag Conserv Dev 6.

[94] Andriamalala G, Gardner CJ (2010) L’utilisation du dina comme outil de gouvernance des ressources naturelles: Leccons tirés de Velondriake, sud-ouest de Madagascar. Trop Conserv Sci 3: 447–472.

[95] Government of Mozambique (2003) Decree No. 43/2003 General Regulation on Marine Fisheries (REPMAR). Decree No 432003.

[96] Swennenhuis J (2011) Strengthening community based fisheries governance in Mozambique: a roadmap developed for IUCN. Gland, Switzerland: International Union for Conservation of Nature. 38 p.

[97] Government of Mozambique (2007) Ministerial Order No. 49/2007 approving the Regulation of the Co-Management Committee on Fishery Sector. 49/2007.

[98] Russo de Sá J (2011) Reflexão Sobre a Gestão Participava Das Pescarias. Maputo, Mozambique: Ministry of Fisheries. 30 p.

[99] Rosendo S, Brown K, Joubert A, Jiddawi N, Mechisso M (2011) A clash of values and approaches: A case study of marine protected area planning in Mozambique. Ocean Coast Manag 54: 55–65 10.1016/j.ocecoaman.2010.10.009

[100] IFAD (2012) Sofala Bank Artisanal Fisheries Project (PPPBAS): Project Completion Report. Completion Report. Rome, Italy: International Fund for Agricultural Development.

[101] IFAD (2010) Republic of Mozambique artisanal fisheries promotion project. Project design document.

[102] National Institute for the Development of Small Scale Fisheries (2012) Co–management in the artisanal fishing sub–sector. Maputo, Mozambique: National Institute for the Development of Small Scale Fisheries (IDPPE).

[103] MRAG (2010) Towards sustainable fisheries management: international examples of innovation. London: MRAG. 93 p.

[104] Wilson J (2012) Beach Seining in Mozambique-Bane or Benefit? IIFET 2012 Tanzania Proceedings. Dar es Salaam, Tanzania.

[105] Garnier J, Silva I, Davidson J, Hill N, Muaves L, et al.. (2008) Co-Management of the Reef at Vamizi Island, Northern Mozambique. CORDIO Status Report. Vol. 2008.

[106] McClanahan TR, Cinner JE, Abunge C (2013) Identifying management preferences, institutional organisational rules, and their capacity to improve fisheries management in Pemba, Mozambique. Afr J Mar Sci 35.

[107] McClanahan TR, Cinner JE, Abunge C, Muthiga N (2012) Identifying management preferences, institutional organizational attributes, and their capacity to improve the management of Pemba, Mozambique fisheries.

[108] Clifton J, Etienne M, Barnes DKA, Barnes RSK, Suggett DJ, et al (2012) Marine conservation policy in Seychelles: Current constraints and prospects for improvement. Mar Policy 36: 823–831 10.1016/j.marpol.2011.11.009

[109] UNDP (2010) Strengthening Seychelles’ protected area system through NGO management modalities.

[110] Hauzer M, Dearden P, Murray G (2013) The effectiveness of community-based governance of small-scale fisheries, Ngazidja island, Comoros. Mar Policy 38: 346–354 10.1016/j.marpol.2012.06.012

[111] Government of South Africa (2012) Policy for the Small Scale Fisheries Sector in South Africa: 56.

[112] Blue Ventures (2012) “Share Your Story”: Madagascar’s First Locally Managed Marine Area (LMMA) Forum. Workshop report. Toliara, Madagascar.

[113] McClanahan TR, Castilla JC, White AT, Defeo O (2008) Healing small-scale fisheries by facilitating complex socio-ecological systems. Rev Fish Biol Fish 19: 33–47 10.1007/s11160-008-9088-8

[114] Syakur A, Wibowo JT, Firmansyah F, Azam I, Linkie M (2012) Ensuring local stakeholder support for marine conservation: establishing a locally-managed marine area network in Aceh. Oryx 46: 516–524 10.1017/S0030605312000166

[115] Cinner JE, McClanahan TR, MacNeil MA, Graham NAJ, Daw TM, et al.. (2012) Comanagement of Coral Reef Social-Ecological Systems. Proc Natl Acad Sci. doi:10.1073/pnas.1121215109. 10.1073/pnas.1121215109 PMC3325732 22431631

[116] McCay BJ, Micheli F, Ponce-Díaz G, Murray G, Shester G, et al (2014) Cooperatives, concessions, and co-management on the Pacific coast of Mexico. Mar Policy 44: 49–59 10.1016/j.marpol.2013.08.001

[117] Auriemma G, Byler K, Peterson K, Yurkanin A, Costello C (2014) A global assessment of Territorial Use Rights in Fisheries to determine variability in success and design. Santa Barbara, California: Bren School of Environmental Science and Management. 132 p.

[118] Guilbeaux M, Parras T, Tan W (2008) Sharing Lessons Learned in Community Marine Management across the Pacific. Workshop report. University of the South Pacific, Fiji: Locally-Managed Marine Area Network. p. 112.

[119] Aalbersberg B, Tawake A, Parras T (2005) Village by village: recovering Fiji’s coastal fisheries. World Resources 2005: The Wealth of the Poor: Managing Ecosystems to Fight Poverty. Washington, D.C: World Resources Institute. 144–152.

[120] The World Bank (2010) World development indicators 2010. [14th ed]. World Bank.

[121] CIESIN (2007) National Aggregates of Geospatial Data: Population, Landscape and Climate Estimates. Palisades, NY: Center for International Earth Science Information Network, Columbia University.

[122] UNDP (2011) Human Development Report 2011: Sustainability and Equity: Towards a Better Future for All.

[123] INSEE (2010) Tableau Économique de Mayotte, Édition 2010. St Denis: Institut National de la Statistique et des Études Economiques.

[124] INSEE (2011) Tableau Économique de La Réunion, Édition 2011. St Denis: Institut National de la Statistique et des Études Economiques.

[125] Statistics South Africa (2012) Census 2011: Census in Brief. Pretoria, South Africa: Statistics South Africa.

[126] Statistics South Africa (2011) Gross domestic product. Annual estimates 2002–2010. Regional estimates 2002–2010. Third quarter 2011. Statistical Release. Pretoria, South Africa: Statistics South Africa.

[127] World Resources Institute (2007) EarthTrends: Environmental Information. Washington DC: World Resources Institute.

