# Supplementary material for: Towards A Network of Locally Managed Marine Areas (LMMAs) in the Western Indian Ocean
Source: PLoS One. 2014 Jul 23;9(7):e103000. doi: 10.1371/journal.pone.0103000 (PMC4108387; doi:10.1371/journal.pone.0103000)
Supplement: Table S1 — (DOCX) [file pone.0103000.s001.docx]

| Table S1: Marine protected areas (level 1 and 2) in the Western Indian Ocean | | | | |
| --- | --- | --- | --- | --- |
| Country | Marine protected area | IUCN Category | Date established | Area (km^2^) |
| Comoros | Mohéli ^6^ | II | 2001 | 404.0 |
| Îles Éparses | Glorieuses ^6^ | IV | 2012 | 43,000.0 |
|  | Ile Tromelin Réserve Naturelle ^6^ | IV | 1975 |  |
|  | Ilot d’Europa Réserve Naturelle ^6^ | IV | 1975 |  |
|  | Ilot de Bassas da India Réserve Naturelle ^6^ | IV | 1975 |  |
| Kenya | Diani | VI | 1995 | 75.0 |
|  | Kisite | II | 1978 | 28.0 |
|  | Kiunga | VI | 1979 | 250.0 |
|  | Malindi | II | 1968 | 6.3 |
|  | Malindi-Watumu | VI | 1968 | 245.0 |
|  | Mombasa Marine National Park | II | 1986 | 10.0 |
|  | Mombasa Marine National Reserve | VI | 1986 | 200.0 |
|  | Mpunguti | VI | 1978 | 11.0 |
|  | Watamu | II | 1968 | 10.0 |
| Madagascar | Kirindy Mitea Marine | II | 2010 | 288.4 |
|  | Masoala ^1^ | II | 1997 | 100.0 |
|  | Nosy Antafana | II | 1989 | 10.0 |
|  | Nosy Hara | Unknown | 2001 | 1,254.7 |
|  | Nosy Tanikely | Unknown | 1966 | 1.8 |
|  | Nosy Ve/Androka | Unknown | 2009 | 820.8 |
|  | Sahamalaza-Nosy Radama | Unknown | 2001 | 127.6 |
|  | Réserve de la Biosphère du Tulear | Not applicable | 2003 |  |
| Mauritius | Anse aux Anglais | Unknown | 2007 | 1.5 |
|  | Balaclava | II | 2000 | 4.9 |
|  | Black River | IV | 2000 | 8.0 |
|  | Blue Bay | Unknown | 2000 | 3.5 |
|  | Grand Bassin | Unknown | 2007 | 14.1 |
|  | Grand Port | IV | 2000 | 18.3 |
|  | Passe Demi | Unknown | 2007 | 7.2 |
|  | Port Louis | IV | 2000 | 3.3 |
|  | Poste Lafayette | IV | 2000 | 2.8 |
|  | Poudre d'Or | IV | 2000 | 25.4 |
|  | Riviere Banane | Unknown | 2007 | 1.5 |
|  | SEMPA | Unknown | 2009 | 43.0 |
|  | Trou d'Eau Douce | IV | 2000 | 5.7 |
| Mayotte | Mayotte ^2^ | Unknown | 2010 | 68,381.0 |
| Mozambique | Bazaruto ^3^ | II | 2001 | 1,430.0 |
|  | North Quirimbas | Unknown | 2008 | 212.0 |
|  | Ponta do Ouro ^4^ | Unknown | 2009 | 678.0 |
|  | Primeiras and Segundas | Unknown | 2012 | 10,409.3 |
|  | Quirimbas | Unknown | 2002 | 1,522.0 |
|  | Vilanculos | Unknown | 2000 | 300.0 |
| Réunion | Réunion | IV | 2007 | 35.0 |
| Seychelles | African Banks | Ib | 1987 | 8.3 |
|  | Aldabra | Ia | 1981 | 142.0 |
|  | Anse Faure Shell Reserve | NA | 1987 | 1.1 |
|  | Aride Island | Ia | 1973 | 0.7 |
|  | Baie Ternay | II | 1979 | 0.9 |
|  | Cousin Island | Ia | 1975 | 0.0 |
|  | Curieuse | II | 1979 | 12.8 |
|  | Ile Cocos, Ile La Fouche, Ilot Platte | Unknown | 1997 | 1.7 |
|  | La Digue Shell Reserve | NA | 1987 | 1.6 |
|  | North East Point Shell Reserve | NA | 1987 | 3.0 |
|  | Port Launay | II | 1979 | 1.5 |
|  | Praslin Shell Reserve | NA | 1987 | 1.7 |
|  | Silhouette | II | 1987 | 16.6 |
|  | Ste. Anne | II | 1973 | 10.0 |
| South Africa | Aliwal Shoal | IV | 2004 | 124.7 |
|  | Maputaland ^5^ | IV | 2000 | 385.2 |
|  | St. Lucia ^5^ | IV | 2000 | 442.7 |
|  | Trafalgar | IV | 2000 | 8.3 |
| Tanzania | Bongoyo Island | II | 1975 | 7.3 |
|  | Fungu Yasini | II | 1975 | 7.5 |
|  | Kiwengwa | Unknown | 2000 | 17.5 |
|  | Mafia Island | VI | 1995 | 615.0 |
|  | Maziwe Island | II | 1981 | 2.6 |
|  | Mbudya Island | II | 1975 | 8.9 |
|  | Mnazi Bay-Ruvuma Estuary | VI | 2000 | 430.0 |
|  | Nyororo, Shungumbili and Mbarakuli | Unknown | 2007 |  |
|  | Pangavini Island | II | 1975 | 2.0 |
|  | Saadani | Unknown | 1969 | 70.0 |
| Tanzania –Zanzibar | Chumbe Island Coral Park (CHICOP) | Ia | 1994 | 0.3 |
|  | Pemba Channel (PECCA) ^6^ | VI | 2005 | 1,000.0 |
|  | Mnemba Island-Chwaka Bay (MIMCA) | VI | 2002 | 0.2 |
| **Total** | **74 MPAs** |  |  | **133,273** |
| 1. Includes 4 MPAs: Tampolo, Masoala-Ambodilaitry, Tanjona and Nose Mangabe 2. Includes Saziley, Passe and N'Gouja 3. Originally gazetted 1971 with 600km^2^ marine area; extended in 2001 4. Includes Ilhas da Inhaca e dos Portugueses 5. Part of iSimangaliso Wetland Park 6. SP and MS had in-depth and more recent experience of these 6 sites and updated the effectiveness ratings from Burke et al [1] accordingly. Mohéli was downgraded from “Effective” to “Partly Effective”; Pemba channel was moved from “Unrated” to “Not Effective”; Glorieuses was moved from “Unrated” to “Partly Effective”; and the other three Îles Éparses reserves (Ile Tromelin, Ilot d’Europa and Ilot de Bassas da India) were moved from “Unrated” to “Effective”. | | | | |

**Reference**

1. Burke L, Reytar K, Spalding M, Perry A (2011) Reefs at risk revisited. Washington, D.C: World Resources Institute. 130 p.
