# Supplementary material for: Towards A Network of Locally Managed Marine Areas (LMMAs) in the Western Indian Ocean
Source: PLoS One. 2014 Jul 23;9(7):e103000. doi: 10.1371/journal.pone.0103000 (PMC4108387; doi:10.1371/journal.pone.0103000)
Supplement: Table S2 — (DOCX) [file pone.0103000.s002.docx]

| Table S2: Locally managed marine areas (level 3 and 4) in the Western Indian Ocean | | | |
| --- | --- | --- | --- |
| Country | Marine protected area | Date established | Area (km^2^) |
| Kenya | Bureni | 2010 | 0.52 |
|  | Jimbo | 2011 | 7.8 |
|  | Kanamai | 2011 | 0.22 |
|  | Kibuyuni | 2010 | 25.1 |
|  | Kiweni | 2010 | 3 |
|  | Majoreni | 2011 | 23.5 |
|  | Mkwakwani/Tradewinds | 2009 | 0.118 |
|  | Mkwiro | 2011 | 8.1 |
|  | Msambweni | 2011 | 0.46 |
|  | Shimoni | 2008 | 3.5 |
|  | Tiwi (Nyari) | 2009 | 0.125 |
|  | Vanga | 2008 | 28.3 |
|  | Wasini | 2008 | 8.6 |
|  | Kuruwitu | 2006 | 0.29 |
| Madagascar | Ambodiforaha | 2011 | 3.1 |
|  | Ambodimangamaro | 2012 | 1.5 |
|  | Amboditangena | 2009 | 1.91 |
|  | Ambodivae bae | 2009 | 200 |
|  | Ambohibola | 2008 | 25 |
|  | Ambondrolava Mangroves | 2008 | 6 |
|  | Analanjahana | 2011 | 2.2 |
|  | Anandrivola | 2012 | 1.5 |
|  | Andakatombaka | 2012 | 1.5 |
|  | Aniribe | 2011 | 2.28 |
|  | Ankarea (Mistio - Tsarabanjina) | 2011 | 1736.9 |
|  | Ankivonjy (Bay de Russes) | 2010 | 1966.66 |
|  | Antsirakivolo | 2009 | 1.86 |
|  | Beheloke | 2008 | 25 |
|  | Belo Sur Mer | 2009 | 200 |
|  | Hoalampano | 2012 | 1.5 |
|  | Imorona | 2009 | 2.09 |
|  | Itampolo | 2008 | 25 |
|  | Mahasoa | 2011 | 2.48 |
|  | Maintimbato | 2009 | 1.91 |
|  | Manjaboaka | 2009 | 240 |
|  | Maromena/Befasy | 2008 | 25 |
|  | Nosy Ve | 2006 | 200 |
|  | Ranobe | 2007 | 243 |
|  | Rantohely | 2009 | 7.24 |
|  | Seranambe | 2012 | 1.5 |
|  | Soariake | 2008 | 750 |
|  | Tahosoa | 2008 | 50 |
|  | Tampolo (Region Cap Est) | 2011 | 3.43 |
|  | Tanandava | 2011 | 2.39 |
|  | Teariake | 2011 | 260 |
|  | Vatolava | 2011 | 1.14 |
|  | Velondriake | 2006 | 640 |
|  | Vohitralanana | 2009 | 3.18 |
| Mozambique | Vamizi Marine Sanctuary ^1^ | 2008 | 18 |
| Tanzania | Boma-Mahandakini ^2^ | 2001 | 145 |
|  | Boza-Sangea ^2^ | 1997 | 396 |
|  | Deepsea-Boma ^2^ | 2000 | 377 |
|  | Mkwaja-Sange ^2^ | 2000 | 388.5 |
|  | Mtang’ata ^2^ | 1997 | 96.5 |
|  | Mwarongo-Sahare ^2^ | 2000 | 195.5 |
|  | Njisopoja ^3^ | 2011 | 884 |
|  | Mbwekieki ^3^ | 2011 | 208 |
|  | Kimsa ^3^ | 2011 | 306 |
|  | Mchimchnumya ^3^ | 2011 | 356 |
|  | Dokichunda ^3^ | 2011 | 478 |
|  | Jojibaki ^3^ | 2011 | 266 |
| Tanzania -- Zanzibar | Menai Bay (MBCA) | 1997 | 470 |
| **Total** | **62 LMMAs** |  | **11,329** |
| 1. Part of North Quirimbas 2. Tanga collaborative Management Areas 3. RuMaKi project | | | |
